# Derivation of a Unique, Algorithm-Based Approach to Cancer Patient Navigator Workload Management

**DOI:** 10.1200/CCI.22.00170

**Published:** 2023-05-19

**Authors:** Xiyitao Zhu, Peng Zhang, Hyojung Kang, Lavanya Marla, Marlene Isabel Robles Granda, Rebecca A. Ebert-Allen, Sarah Stewart de Ramirez, Tenille Oderwald, Mackenzie McGee, Jonathan A. Handler

**Affiliations:** ^1^ University of Illinois at Urbana-Champaign, Champaign, IL; ^2^ OSF HealthCare System, Peoria, IL; ^3^ University of Illinois College of Medicine at Peoria, Peoria, IL; ^4^ Department of Emergency Medicine, Northwestern University Feinberg School of Medicine, Chicago, IL

## Abstract

**PURPOSE:**

Cancer patient navigators (CPNs) can decrease the time from diagnosis to treatment, but workloads vary widely, which may lead to burnout and less optimal navigation. Current practice for patient distribution among CPNs at our institution approximates random distribution. A literature search did not uncover previous reports of an automated algorithm to distribute patients to CPNs. We sought to develop an automated algorithm to fairly distribute new patients among CPNs specializing in the same cancer type(s) and assess its performance through simulation on a retrospective data set.

**METHODS:**

Using a 3-year data set, a proxy for CPN work was identified and multiple models were developed to predict the upcoming week's workload for each patient. An XGBoost-based predictor was retained on the basis of its superior performance. A distribution model was developed to fairly distribute new patients among CPNs within a specialty on the basis of predicted work needed. The predicted work included the week's predicted workload from a CPN's existing patients plus that of newly distributed patients to the CPN. Resulting workload unfairness was compared between predictor-informed and random distribution.

**RESULTS:**

Predictor-informed distribution significantly outperformed random distribution for equalizing weekly workloads across CPNs within a specialty.

**CONCLUSION:**

This derivation work demonstrates the feasibility of an automated model to distribute new patients more fairly than random assignment (with unfairness assessed using a workload proxy). Improved workload management may help reduce CPN burnout and improve navigation assistance for patients with cancer.

## INTRODUCTION

Patient navigators guide patients through the health care system. This includes help going through the screening, diagnosis, treatment, and follow-up of a medical condition, such as cancer.^1^ Cancer patient navigators (CPNs) can decrease the time from diagnosis to treatment.^2,3^ CPNs can be professionals, paraprofessionals, or community laypeople.^4^

**CONTEXT**

**Key Objective**
Can a machine-learned algorithm outperform random assignment in fairly distributing patients among multiple cancer patient navigators (CPNs) working in the same cancer specialty to even out workloads?
**Knowledge Generated**
Our CPNs usually distribute new patients on a simple alternating basis, a nearly random approach. This retrospective simulation study on a real-world data set demonstrated that the algorithm more fairly distributes patients among CPNs within a cancer specialty than random distribution.
**Relevance**
CPNs often suffer from work overload and have been shown to have high risk for burnout. Better workload management may reduce CPN burnout and lead to more effective and efficient navigation assistance for patients with cancer, allowing greater scalability of this vital resource to all oncology patients in need, regardless of geography.

Despite demonstrated efficacy and growing demand, CPN programs are often underfunded,^5^ causing understaffing that increases the potential frequency and severity of CPN work overload. Navigation has additional challenges for American rural populations who tend to live farther from health care facilities and to have older age, higher poverty rates, less health care access, and less health insurance.^6-8^

Some institutions use general purpose CPNs; others use specialized CPNs (each CPN manages patients with certain cancer types). Our system has specialized oncology nurse navigators serving a mixed rurality population. Except in unusual cases, patients are distributed to relevant specialized CPNs on an alternating basis. Anticipated patient needs, CPN experience, and CPN existing workload are generally not considered for this distribution. Thus, we distribute patients nearly randomly among specialized CPNs. CPN workloads vary widely over time. One CPN in a specialty can be overloaded, whereas the others have lighter workloads. To maintain patient relationships, CPNs almost never balance workloads by reassigning patients to other CPNs. Periods of heavy workload can create tremendous stress and high risk of burnout. CPN burnout has been reported as a serious problem in real-world oncology practice.^9^ Our CPN leadership believes that better workload management could improve patient care. Therefore, we sought to create an algorithm that distributes new patients more fairly, taking into account the work needed for new patients and also those already in each CPN's panel, and then to assess its performance through simulated distribution on a real-world, retrospective data set.

## METHODS

This study was approved by OSF Clinical Research and granted exempt status by the University of Illinois College of Medicine at Peoria Institutional Review Board.

### Data Set

Included patients had at least one interaction during a 3-year period with the 13 specialty CPNs operating at our health system's largest hospital. The period was chosen to provide enough data to train and assess a model while using recent enough data to be relevant to current practice. The last few patient-weeks of data were removed to ensure that the target variable was populated, resulting in a 150-week data set. An interaction was defined as the writing or editing of a note or care plan or creation of a CPN encounter in the electronic health record (EHR). The data set contained one row per patient per day. Patients were considered active between their first CPN interaction and 90 days after their last CPN interaction. Since the model simulated weekly patient distribution, only Mondays were retained.

For model development (described below), patients were randomly split into training (80%) and test (20%) sets, allocated so that all rows for any patient would be in the same set.

The data set contained daily updates about every patient's clinical state and level of complexity for use in predicting the near-term workload for every patient at any point in time. Dozens of input features were used to make each prediction each week for every patient, including demographic, health care utilization, clinical, date-related, and other data elements (eg, trends; Data Supplement [Section 1]). All inputs were relative to the effective date of the row. Socioeconomic status was estimated by the Area Deprivation Index (ADI),^10,11^ and rurality was estimated using Rural-Urban Continuum Codes (RUCC),^12^ each assigned using the patient's home zip code. Since the ADI provides values for nine-digit zip codes, the average ADI for all nine-digit zip codes in the five-digit zip code was used.

Features with null values in 80% or more of the rows were removed. Medical insurance was combined into six categories: Medicare, Medicaid, self-pay, commercial except Blue Cross/Blue Shield, Blue Cross/Blue Shield, and others. Missing insurance values were imputed to Others. Missing ADI and RUCC values were replaced by their training set means.

We evaluated potential proxy metrics for CPN work, including the number of notes written (notct); the number of times notes were either created or edited (notct2); the number of encounters per day created in the EHR, counting not more than one encounter per patient per day (enct); and the daily time difference in first and last EHR interactions of any kind in the audit log (dur). For each, the average value per day over a month was compared with the number of active patients in a CPN's panel during that month. enct was chosen as the workload metric proxy because its Pearson correlation^13^ with panel size was most consistently higher than that of the other candidate metrics (Data Supplement [Section 2]).

### Unfairness Measurement

**FIG 1.**
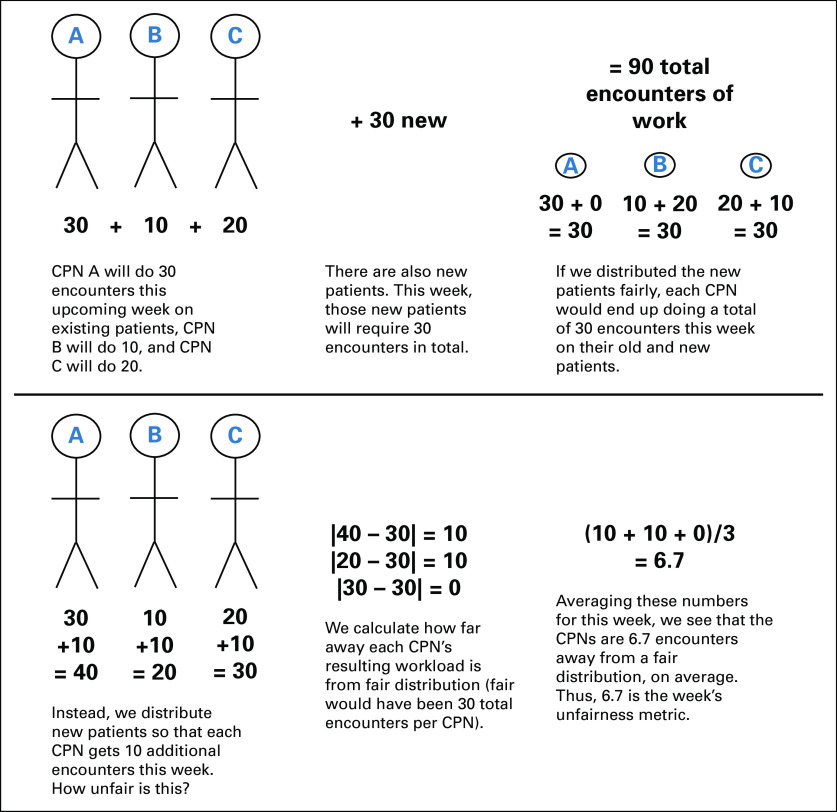
Example calculation of the week's unfairness metric. CPN, cancer patient navigator.

We sought to enhance workload fairness among CPNs within a specialty. Each week, we calculated an unfairness metric for each CPN specialty by taking the average of the absolute magnitude of the differences between a perfectly fair distribution of work and the actual distribution of work that week among the CPNs in the specialty (Fig 1).

We aimed to minimize the magnitude of the unfairness metric each week, reducing the unfairness resulting from the work generated by patients each week *for as long as each patient was active with a CPN*.

### System Overview

We developed a model to predict the work that will be needed by every active patient in the upcoming week. Then, we built a model that uses those predictions as an input to more fairly distribute new patients among CPNs of the same specialty.

### The Prediction Model

A two-stage system predicts the work that each patient (both new and existing) will require in the ensuing week. First, an unsupervised clustering model automatically identifies patients with similar characteristics (clusters) and assigns each patient each week to a cluster. Then, a regression model predicts the work required for each patient each week, using the cluster identifier as one of its input features.

To develop the clustering model, we used all input fields except day count since last diagnosed cancer. We applied unsupervised k-means and k-prototype algorithms^14,15^ on our training set. For k-means, one-hot encoding/multiple correspondence analysis was applied for categorical features. K-prototype was chosen because of its ability to handle different feature types, support for explainability, and consistently better performance than k-means. The number of clusters (7) was determined using grid search and elbow methods (Data Supplement [Section 5]). Each patient-week was assigned a cluster on the basis of its input features, so a patient could be assigned to different clusters on different weeks.

Then, we developed three supervised regression models, each one built from one of the most common and successful open-source machine learning libraries^16^ (Neural Network,^17^ Random Forest,^18^ and XGBoost^19^). The models use the data that would have been available at prediction time from the many input features to make regular (eg, weekly), individualized predictions of near-term work that will be needed for each and every active patient (both existing and new). Each model used all input features except days since last CPN interaction and days since last cancer diagnosis and added the cluster identifier as an input feature. Model performance was enhanced through random grid search hyperparameter tuning (Data Supplement [Section 3]). For computational efficiency, the random grid search used 15 random hyperparameter combinations from the search space. Three-fold cross-validation was applied over the training set to identify the hyperparameter combination achieving the smallest mean squared error (MSE). Each model's best hyperparameter combination was retained, and each model made predictions on the test data set. The MSE for the predicted and actual number of patient-CPN encounters in the next week served as the evaluation metric. MSE values for predictors were 0.103 for XGBoost, 0.114 for Random Forest, and 0.129 for Neural Network. Having the lowest MSE, the XGBoost predictor was selected for ongoing use.

### The Distribution Model

An optimization model was developed to distribute patients among CPNs in each specialty, with the goal of equalizing the week's workload among CPNs of the same specialty (Data Supplement [Section 4]). The unfairness metric served as the objective function of the model. Our program seeks to maintain the patient-CPN relationship, so the only consistency constraint imposed was on allocations to ensure that patients remained with their initially assigned CPN throughout their time in the panel. This prediction-informed distribution approach was applied to the test set, and the mean and standard deviation (SD) of the resulting weekly unfairness metric values were calculated.

### Assessing Distribution Performance

As a retrospective derivation study, we did not have reliable historical CPN patient assignments, hiring, and work schedules to use for comparison. Therefore, we performed a simulation on our real-world data set. CPNs were simulated as working at full capacity each week, the number of CPNs for each specialty was based on the number of CPNs working in that specialty when the data set was gathered, and only specialties having more than one CPN were included (Table 1).

**TABLE 1.**
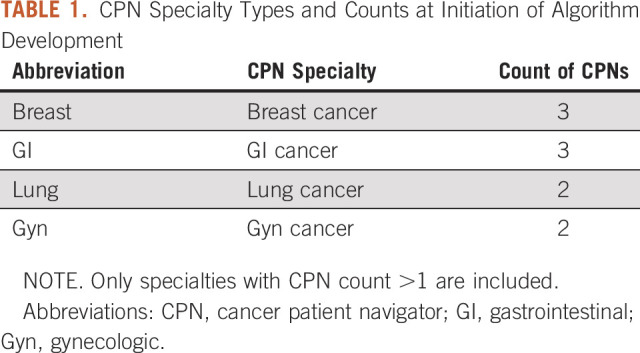
CPN Specialty Types and Counts at Initiation of Algorithm Development

Since patient distribution among CPNs at our institution approximates random distribution (as noted earlier), we used random distribution of patients to represent the expected performance of our current distribution methodology. From the test set, each week's new patients were randomly distributed among the CPNs in the relevant specialty and the unfairness value was calculated. To ensure that results were representative of expected performance, each week's random distribution was repeated 10,000 times, and the mean of those unfairness values was used for that week. This was repeated for each of the 150 weeks, and the mean and SD of those 150 values were calculated.

Similarly, the prediction-informed model distributed each week’s new patients from the 150-week test set, taking into account the predicted workload from each CPN’s existing patients and the predicted workload needed by the new patients. The unfairness value was calculated for each week, and a mean and SD were calculated for the resulting values.

For both models, we computed 95% CIs for the weekly unfairness metric using the following formula: mean ± 1.98 × (SD/square root of n).^20,21^

The Data Supplement (Section 6) contains the methodology and results for a future-informed distribution approach comparison.

## RESULTS

The data set contained 273,057 records comprising 13,033 unique patients. Demographics are summarized in Table 2.

**TABLE 2.**
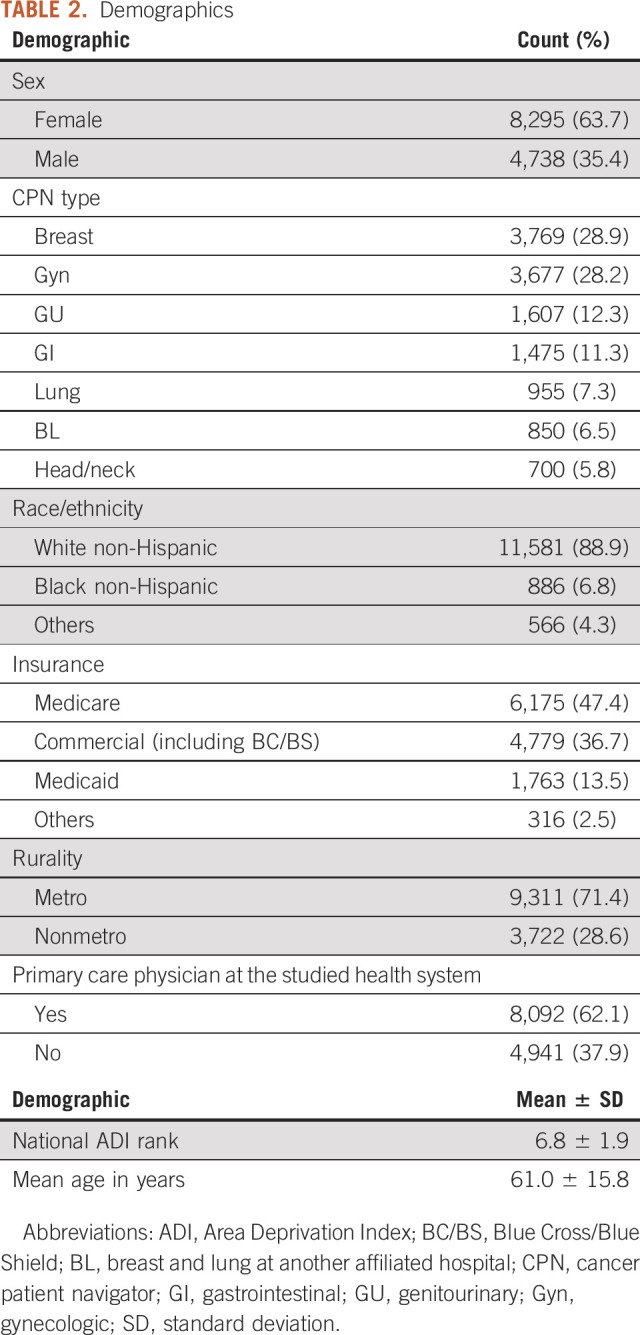
Demographics

Workload variability was quantified using the workload proxy metric, with CPN encounters per patient per month (30 days) ranging from 0 to 15, having a median of 0.0 and a mean and SD of 0.5 ± 1.1. The resulting coefficient of variation was 220%, and the skew was 1.36, demonstrating that the work across patients and time were highly variable and positively skewed (ie, a long tail of patients needing much more work than the average patient).

**FIG 2.**
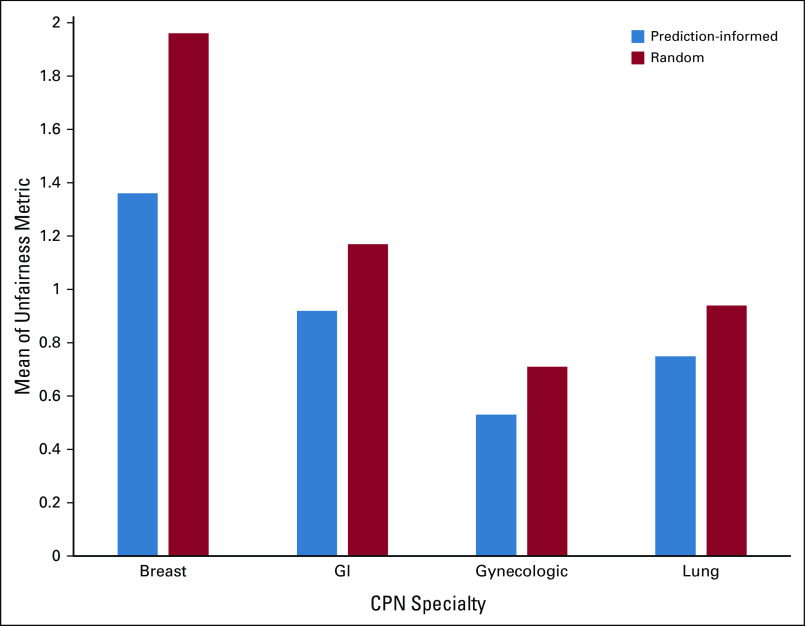
Performance of each distribution method for each CPN specialty. The y-axis is the mean of the unfairness metric over the 150 weeks covered by the test data set. Lower values are better. CPN, cancer patient navigator.

Tables 3 and 4 provide performance metrics for the distribution approaches, and Figure 2 shows comparative performance graphically. A smaller unfairness metric value indicates less overall unfairness (greater fairness). Prediction-informed distribution significantly outperformed random allocation for all CPN specialties, with lower means and nonoverlapping 95% CIs.

**TABLE 3.**
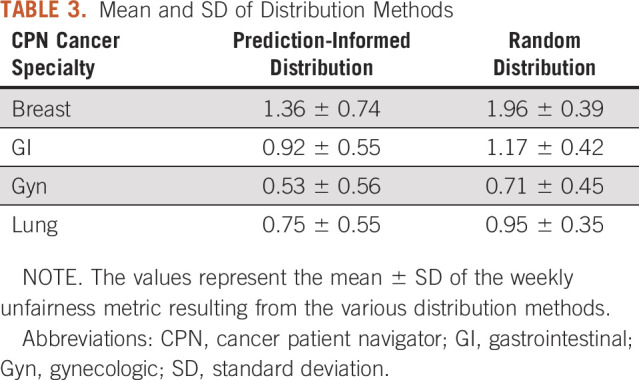
Mean and SD of Distribution Methods

**TABLE 4.**
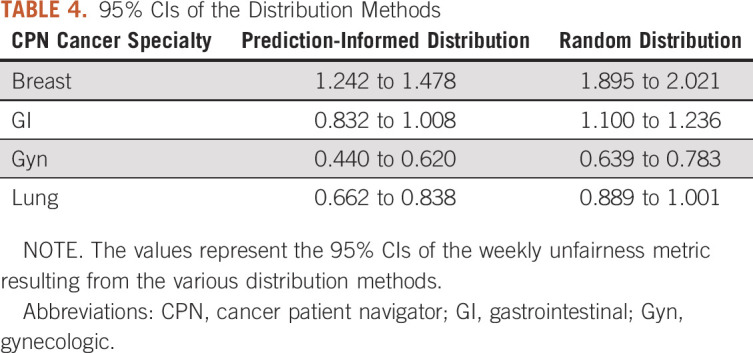
95% CIs of the Distribution Methods

## DISCUSSION

To our knowledge, this work may represent the first-ever description of an automated, algorithm-driven approach to even out CPN workloads. Optimization has been applied to health care staffing and patient allocation in other health care domains,^22-25^ but this is usually applied to shifts rather than individuals. Previous work has demonstrated that lay patient navigators may accurately predict the work intensity needed for a patient. However, the navigators predicted total anticipated navigation time during the entire period that the patient would be followed by the CPN, not the anticipated work required in the immediate future of a given date. Frequent, near-term predictions, like those generated by our system, are needed to achieve near-continuous improvements in workload fairness. In addition, the predictions were only made after the navigator and patient connected to complete an assessment of anticipated needs.^26^ Various tools may also predict the CPN work that a patient will need, but again, they often require the navigator to first connect with the patient to gather needed information.^27^ These approaches do not offer an optimal solution for navigation programs that seek to avoid transferring care among CPNs once relationships have been established. A PubMed search returned no previous studies of automated, algorithm-driven patient distribution using already-existing information to even out CPN workloads.^28^

CPN programs increase access to vital services for patients with cancer, help distribute work among busy oncology care teams, and can improve patient outcomes.^29^ Navigation has been shown to improve patient satisfaction while also demonstrating financial benefits for cancer programs. Therefore, these programs may continue to grow.^5^ Balancing a growing workload across CPNs to protect fairness and avoid burnout comes with substantial challenges, perhaps especially for programs serving rural populations.

Our health care system serves a mixed-rurality population. Workloads must be fairly distributed to give each CPN enough time to serve the significant needs of this population. Allocating CPN resources can be even more challenging in systems that serve rural populations as they have higher cancer death rates, lower utilization of cancer-relevant preventive services, lower likelihood of receiving guideline-recommended treatment, higher travel times and costs to get to health care sites, higher poverty rates, and reduced access to translation resources.^30-33^

Given this, we sought a method to more fairly distribute patients to achieve equitable CPN workloads to help prevent burnout commonly experienced by CPNs^9^ and to facilitate effective navigation. We built a prediction-informed model to distribute new patients. The model predicts the near-term work that will be generated by each new patient and each patient already followed by the CPNs, and then it distributes the new patients to minimize differences between CPNs in their total predicted workload. Our institution currently distributes patients among CPNs using a mostly random method, and this work shows that prediction-informed distribution significantly outperformed random distribution for all studied CPN specialties.

The system focuses on distributing new patients because CPNs do not transfer their existing patients to other CPNs when workloads become overwhelming. They prefer to retain their patients for the laudable purpose of maintaining a consistent patient-CPN relationship. Therefore, the algorithm uses the only lever available to equalize workloads: the distribution of new patients.

Our data set had an average of 13.4 new patients per week needing distribution in specialties with two CPNs and 20.1 patients per week in specialties with three. However, those averages severely understate the challenge of distribution. They do not reflect the increasing patient volumes over time, the high variability in the number of new patients each week that can lead to much higher numbers to distribute, or the high variability of work required by different patients at different times (demonstrated in our results). Even more significantly, the magnitude and variability of workload do not only come solely from new patients but also come from the ongoing and evolving work needs of patients that the CPN was already managing (which can exceed 200 in some cases). The near-term work must be predicted for every active patient in a cancer specialty—both new and existing—to distribute new patients in a manner that evens out anticipated CPN workloads. Our system uses individualized, updated patient predictions to automatically and effectively perform this nontrivial task with each distribution.

The prediction-informed model already significantly outperforms the random distribution approach that approximates our current distribution methodology. Future work may further improve the performance of the predictive model, leading to even greater fairness in automated distribution. For example, the ADI input feature implicitly assumes that all within a geographic area have equal disadvantage and the averaging of ADI values in a five-digit zip code further groups socioeconomic levels. More individualized socioeconomic indicators and/or entirely new input features (eg, using natural language processing^34,35^) might lead to an even more effective algorithm.

Our CPN task durations are not captured in structured data. Although we tested several proxies for CPN workload to find the best fit, our proxy is likely imperfect. We also assumed, potentially inaccurately, that all work done by a CPN during the study period was performed for CPN purposes.

At institutions that use patient information, CPN abilities, and/or CPN workloads to inform patient distribution, our model might have less standalone utility. However, it may provide additional information to distribute patients with even greater fairness.

Although performed on a real-world data set, this retrospective simulation could not assess whether the model improves CPN satisfaction or reduces CPN burnout. This work was developed for a mixed-rurality population and a cancer site–specialized oncology nurse navigator program and may not generalize to other contexts.

Our simulation used weekly patient distribution (to facilitate computation), but the model can distribute at any frequency (including daily). Although not tested, more frequent distribution might have even better performance by enabling finer workload adjustment. The simulation also assumed that the number of CPNs within a specialty did not vary. However, the model can accommodate varying CPN numbers, and the model outperformed random distribution regardless of whether the specialty had two or three CPNs.

Random assignment, rather than actual CPN assignment, was used for comparison. Our actual manual distribution method approximates random assignment because our CPNs usually take turns picking up patients without regard to patient characteristics, CPN experience, or existing CPN workload. With that said, our distribution is not perfectly random because an overworked CPN may occasionally skip a turn in the rotation. This would not be replicated in the simulation, and our actual patient distribution may be better than random at times. However, since skipping a turn is unusual and prediction-informed distribution had unfairness means about 20%-30% lower than random, our simulation suggests that the system holds promise and that a prospective study is warranted to confirm its utility.

This foundational work describes the modeling framework, demonstrates the feasibility, and provides the preliminary evidence needed to support and inform a more definitive prospective trial not subject to these limitations.

In conclusion, in this retrospective derivation, feasibility, and preliminary validation study, prediction-informed distribution outperformed random assignment in achieving equitable workloads across CPNs within a specialty, as measured by a workload proxy. Better workload management may reduce CPN burnout and lead to more effective and efficient navigation assistance for patients with cancer, allowing greater scalability of this vital resource to all oncology patients in need, regardless of geography.

## References

[1] National Cancer Institute: Definition of patient navigator. NCI Dictionary of Cancer Terms, National Cancer Institute. https://www.cancer.gov/publications/dictionaries/cancer-terms/def/patient-navigator

[2] Krok-Schoen JL, Oliveri JM, Paskett ED: Cancer care delivery and women’s health: The role of patient navigation. Front Oncol 6:2, 2016 26858934 10.3389/fonc.2016.00002 PMC4729879

[3] Enomoto LM, Fenstermaker J, Desnoyers RJ, et al: Oncology navigation decreases time to treatment in patients with pancreatic malignancy. Ann Surg Oncol 26:1512-1518, 2019 30652224 10.1245/s10434-019-07157-6

[4] Braun KL, Kagawa-Singer M, Holden AEC, et al: Cancer patient navigator tasks across the cancer care continuum. J Health Care Poor Underserved 23:398-413, 2012 22423178 10.1353/hpu.2012.0029 PMC3302357

[5] Kline RM, Rocque GB, Rohan EA, et al: Patient navigation in cancer: The business case to support clinical needs. JCO Oncol Pract 15:585-590, 2019 10.1200/JOP.19.00230 PMC8790714 31509483

[6] National Rural Health Association: About Rural Health Care. NRHA. https://www.ruralhealthweb.org/about-nrha/about-rural-health-care

[7] CDC: Leading causes of death in rural America. Centers for Disease Control and Prevention. https://www.cdc.gov/ruralhealth/cause-of-death.html

[8] Golembiewski EH, Gravholt DL, Torres Roldan VD, et al: Rural patient experiences of accessing care for chronic conditions: A systematic review and thematic synthesis of qualitative studies. Ann Fam Med 20:266-272, 2022 35606138 10.1370/afm.2798 PMC9199043

[9] Dean M, Gentry E, Gentry S: Caring for the caregiver: Using a tool to increase navigator self-awareness/self-examination regarding compassion fatigue. J Oncol Navig Surviv 12:404, 2021

[10] University of Wisconsin School of Medicine and Public Health: 2019 Area Deprivation Index v3.0. https://www.neighborhoodatlas.medicine.wisc.edu/

[11] Kind AJH, Buckingham W: Making neighborhood disadvantage metrics accessible: The Neighborhood Atlas. N Engl J Med 378:2456-2458, 2018 29949490 10.1056/NEJMp1802313 PMC6051533

[12] USDA Economic Research Service: Rural-urban continuum codes. https://www.ers.usda.gov/data-products/rural-urban-continuum-codes/documentation/

[13] The Pandas Development Team: pandas.DataFrame.corr—pandas 1.4.3 documentation. https://pandas.pydata.org/docs/reference/api/pandas.DataFrame.corr.html

[14] Python Software Foundation: Python. https://www.python.org/about/

[15] de Vos NJ: kmodes: Python implementations of the k-modes and k-prototypes clustering algorithms for clustering categorical data. https://github.com/nicodv/kmodes

[16] Goldbloom A: What algorithms are most successful on Kaggle? https://kaggle.com/code/antgoldbloom/what-algorithms-are-most-successful-on-kaggle

[17] Keras Team: Keras documentation: About Keras. https://keras.io/about/

[18] sklearn.ensemble.RandomForestRegressor. scikit-learn. https://scikit-learn/stable/modules/generated/sklearn.ensemble.RandomForestRegressor.html

[19] XGBoost documentation—XGBoost 1.7.0 documentation. https://xgboost.readthedocs.io/en/stable/

[20] Lane DM: Confidence interval for the mean, in Lane DM (Project Leader), Online Statistics Education: An Interactive Multimedia Course of Study. Houston, TX, Rice University. http://onlinestatbook.com/

[21] Lane DM: Inverse t distribution, in Lane DM (Project Leader), Online Statistics Education: A Multimedia Course of Study. Houston, TX, Rice University. https://onlinestatbook.com/2/calculators/inverse_t_dist.html

[22] Malkoske KE, Sixel KE, Hunter R, et al: COMP report: An updated algorithm to estimate medical physics staffing levels for radiation oncology. J Appl Clin Med Phys 22:6-15, 2021 10.1002/acm2.13364 PMC8364262 34318570

[23] Meyer KR, Fraser PB, Emeny RT: Development of a nursing assignment tool using workload acuity scores. J Nurs Adm 50:322-327, 2020 32427663 10.1097/NNA.0000000000000892 PMC8402942

[24] Abadi MQH, Rahmati S, Sharifi A, et al: HSSAGA: Designation and scheduling of nurses for taking care of COVID-19 patients using novel method of Hybrid Salp Swarm Algorithm and Genetic Algorithm. Appl Soft Comput 108:107449, 2021 33967657 10.1016/j.asoc.2021.107449 PMC8086267

[25] Debats CEJM, Dellaert NP, Pouwels S, et al: Balancing workload in the PACU by using an integrated OR planning methodology. J Perianesth Nurs 36:279-290, 2021 33622613 10.1016/j.jopan.2020.09.004

[26] Carroll JK, Winters PC, Purnell JQ, et al: Do navigators’ estimates of navigation intensity predict navigation time for cancer care? J Cancer Educ 26:761-766, 2011 21556957 10.1007/s13187-011-0234-y PMC4401038

[27] Strusowski T: Key considerations for an evidence-based oncology patient navigation–specific acuity tool: A scoping review, 2019. https://www.jons-online.com/issues/2019/july-2019-vol-10-no-7/2479-key-considerations-for-an-evidence-based-oncology-patient-navigation-specific-acuity-tool-a-scoping-review

[28] (patient navigator OR cpn OR onn OR nurse navigator OR oncology navigator) AND (cancer OR oncology) AND (caseload OR work OR workload) AND algorithm—Search Results—PubMed. https://pubmed.ncbi.nlm.nih.gov/?term=%28patient+navigator+OR+cpn+OR+onn+OR+nurse+navigator+OR+oncology+navigator%29+AND+%28cancer+OR+oncology%29+AND+%28caseload+OR+work+OR+workload%29+AND+algorithm

[29] Qian F, Gates M, Bisner S, et al: Benefits, cost, and activities of patient navigation (PN) program for colorectal cancer screening at the Charles B. Wang Community Health Center (CBWCHC). J Immigr Minor Health 22:476-483, 2020 31254139 10.1007/s10903-019-00913-6

[30] Atkins GT, Kim T, Munson J: Residence in rural areas of the United States and lung cancer mortality. Disease incidence, treatment disparities, and stage-specific survival. Ann Am Thorac Soc 14:403-411, 2017 28118039 10.1513/AnnalsATS.201606-469OC

[31] Zahnd WE, James AS, Jenkins WD, et al: Rural-urban differences in cancer incidence and trends in the United States. Cancer Epidemiol Biomarkers Prev 27:1265-1274, 2018 28751476 10.1158/1055-9965.EPI-17-0430 PMC5787045

[32] Keim-Malpass J: Identifying barriers to navigation needs of cancer survivors in rural areas. https://www.jons-online.com/issues/2015/october-2015-vol-6-no-5/1357-identifying-barriers-to-navigation-needs-of-cancer-survivors-in-rural-areas

[33] Cole AM, Jackson JE, Doescher M: Colorectal cancer screening disparities for rural minorities in the United States. J Prim Care Community Health 4:106-111, 2013 23799717 10.1177/2150131912463244

[34] Bako AT, Taylor HL, Wiley K, et al: Using natural language processing to classify social work interventions. Am J Manag Care 27:e24-e31, 2021 33471465 10.37765/ajmc.2021.88580 PMC8005360

[35] Sarker A, Al-Garadi MA, Yang YC, et al: Defining patient-oriented natural language processing: A new paradigm for research and development to facilitate adoption and use by medical experts. JMIR Med Inform 9:e18471, 2021 34581670 10.2196/18471 PMC8512184

